# Supplementary material for: Microbiota Co-Metabolism Alterations Precede Changes in the Host Metabolism in the Early Stages of Diet-Induced MASLD in Wistar Rats
Source: Int J Mol Sci. 2025 Feb 2;26(3):1288. doi: 10.3390/ijms26031288 (PMC11818068; doi:10.3390/ijms26031288)
Supplement: Supplementary file 1 [file ijms-26-01288-s001.zip › ijms-3434087-supplementary.pdf]

Supplementary information

Article

Microbiota Co-Metabolism Alterations Precede Changes in the Host Metabolism in the Early Stages of Diet-Induced MASLD in Wistar Rats

María Martín-Grau [1,2,*], Pilar Casanova [1,2], Laura Moreno-Morcillo [1,2], José Manuel Morales [1,2], Vannina G. Marrachelli [2,3] and Daniel Monleón [1,2,*]

[1] Departament de Patologia, Universitat de València, 46010 Valencia, Spain; pilar.casanova@uv.es (P.C.); laura.moreno-morcillo@uv.es (L.M.-M.); j.manuel.morales@uv.es (J.M.M.)
[2] INCLIVA Biomedical Research Institute, 46010 Valencia, Spain
[3] Departament de Fisiologia, Universitat de València, 46010 Valencia, Spain; vannina.gonzalez@uv.es
* Correspondence: maria.martin-grau@uv.es (M.M.-G.), and daniel.monleon@uv.es (D.M.)

List of contents:

- **Material and methods supplementary data (page 2)**
- **Supplementary figures and tables (page 5)**

S4. Material and methods

S4.1. Animals and housing – IP GTT – Postmortem procedures

Animals were fed with CTL diet (67% kcal from carbohydrate, 13% kcal from fat, 20% kcal from protein; energy density of 2.9 Kcal/g; 2014S, ENVIGO, West Lafayette, Indiana, USA) or 45 % HFD (40.7% kcal from carbohydrate, 44.6% kcal from fat, 14.7% kcal from protein; energy density of 4.7 Kcal/g; TD.08811, Ssniff, Soest, Germany) for 21 weeks. The energy intake was calculated from the energy density from each diet.

IP GTTs were performed at week 12 (t12) and week 21 (t21). The animals were fasted at least 4 hours before starting. Basal glycaemia (mg/dL) was measured before the injection of glucose in the saphenous veins of the left hind leg using a glucometer and test strips (AccuChek Aviva, Roche Diabetes Care Spain, Barcelona, Spain). Then, a glucose dose of 2g of glucose/Kg of animal (Glucocemin 33%, Braun, Kronberg, Germany) was injected to each rat. Afterwards, basal glycaemia was measured again at 15, 30, 60, and 120 min after the injection of glucose. For data processing, basal glycaemia value obtained at the starting point (0 min) was subtracted from each value at each time point (15, 30, 60 and 120 min). Finally, the area of the curve (AOC) was calculated by MATLAB software (MATLAB R2014a, MathWorks, Natick, MA, USA) to normalize all the values.

After 21 weeks, animals were sacrificed by inhalation of 5% isoflurane. Blood samples were collected during the sacrifice and two aliquots were acquired, one containing plasma (preserved in Ethylenediaminetetraacetic acid (EDTA), 0.4 M and pH 8, E6511-100G, Sigma, San Luis, Misuri, USA) and the other containing serum (without EDTA). Plasma was used for biochemical analysis at t21, while serum was designated for metabolomics at t21 and stored at -80°C until analysis. Furthermore, the livers were collected during the sacrifice. Initially, they were weighted and subsequently, they were sectioned, and preserved in various reagent based on their intended use. For histological examination, a segment was fixed in 4% formaldehyde (11699455, VWR Q-Path Chemicals, Barcelona, Spain) for a minimum of 48h and then embedded in paraffin for paraffin-embedded histological analysis. Another portion was submerged in tissue-Tek O.C.T compound (4583, Sakura, Japan) and frozen at -80 °C until the cutting process for non-paraffin-embedded histological analysis. The remainder of the organ was frozen at -80°C for subsequent metabolomic analysis.

S4.2. Metabolomics using Proton Nuclear Magnetic Resonance (^{1}H-NMR)

S4.2.1. Sample preparation

S4.2.1.1. Liquid biological samples

For serum, urine, and faecal extract, 20 µL of mix (sample + TSP/buffer) were introduced into 1mm NMR capillary tube (Z107504, Bruker, Karlsruhe, Germany) and measured by the 1mm triple resonance (TXI) probe of ^{1}H-NMR.

Serum was obtained from the whole blood after clotting and serial centrifugations at 2500g. Aliquots of 25 µL of serum were frozen at -80 °C until its use. Defrosted samples were mixed with 3.75 µL of 3-(trimethylsilyl)-2,2,3,3-tetradeuteropropionic acid (TSP) (11202, Deutero, Kastellaun, Germany) to obtain a final concentration of 2.5 mM. Only 20 µL of the mix was pipetted into 1mm NMR capillary tube (Z107504, Bruker, Karlsruhe, Germany).

Urine samples were defrosted and centrifuged at 14800 rpm for 5 min. In some samples, a pellet appeared, and was discarded. For ^{1}H-NMR, 20 µL of clear urine supernatant were used. For stool samples, a previous aqueous extraction was carried out. For this, 60 mg of solid sample was mixed with 100 µL of distilled water. The samples were centrifuged at 14800 rpm for 5 min. Then, they were resuspended and frozen at -80 °C for 5 min, heated at 37°C for 3 min, and centrifuged again at 14800 rpm for 5 min. The samples were then resuspended and centrifuged again at 14800 rpm for 5 min. The supernatant was collected and 20 µL of it were used for ^{1}H-NMR.

In addition, a phosphate buffer composed of 1.5 M of KH_2PO_4 (795488, Sigma-Aldrich, Germany), and 5.8 mM of TSP was used for adjusting the pH of urine and faecal extracts. Phosphate buffer was made in sterile deuterium oxide (D_2O) (1.13366, Sigma, San Luis, Misuri, USA) and adjusted to pH 7.4 with potassium hydroxide (KOH) (P/5600/53, Fisher Scientific, Waltham, MA, USA). The phosphate buffer was kept cold at 4°C. Before its use, its pH was checked, and it was centrifuged at 14800 rpm for 3 min. Then, a reaction mix with 20 µL of sample (urine or faecal extraction) and 4 µL of phosphate buffer was prepared (being the final concentration of TSP 1 mM). The mix was left at 4°C for 15 min. After incubation, the mix was centrifuged at 14800 rpm for 3 min. Finally, only 20 µL of the mix was pipetted into the 1mm NMR capillary tube (Z107504, Bruker, Karlsruhe, Germany).

S4.2.1.2. Tissue samples

Livers stored at -80°C were fractionated with liquid nitrogen. Fragments were collected, weighed (between 50-60 mg), and introduced into a zirconia rotor (HZ07213, Bruker, Karlsruhe, Germany). Then, 40 µL of D_2O were added and the rotors were sealed. The rotor was manually introduced into the ^{1}H-NMR equipment. The High-Resolution Magic Angle Spinning (HR-MAS) ^{1}H-NMR probe was used for the measurement. The spinning frequency was 5 kHz and the temperature of measurement was 277 K (~4°C). The liver fragment mass was considered for the subsequent normalization of the spectra.

S4.2.2. ^{1}H-NMR spectra analysis

A Bruker AVANCE III Nuclear Magnetic Resonance (NMR) spectrometer (Bruker BioSpin GmbH, Rheinstetten, Germany) operating at 600.13 MHz proton (^{1}H) – NMR frequency was used. Once the samples were measured by the NMR spectrometer, the spectra were obtained. Firstly, MestReNova software (MestReNova v14.1.1, Mestrelab Research S.L, Santiago de Compostela, Spain) was used to correct the phase, the baseline, and the reference guide of the spectra. Secondly, Chenomx software (Chenomx NMR Mixture Analysis v8.1, Edmonton, Canada) was used to assign the different metabolites to each peak of the spectra. Data bases such as PubMed, Human Metabolome Database (HMDB), and Kyoto Encyclopaedia of Genes and Genomes (KEGG) were also used to verify metabolites. The listed metabolites appear in **Tables S1-S4**. Thirdly, MATLAB software (MATLAB R2014a, MathWorks, Natick, MA, USA) was used to obtain the Partial Least-Squares Discriminant Analysis (PLS-DA) model and the Variable Importance in the Projection (VIP) scores. Only metabolites with a VIP score higher than 1 were considered as metabolites which contribute to the group distribution in the PLS-DA. Mean per animal group, standard deviation, and relative fold change of each metabolite were calculated. The formula applied to obtain the relative fold change was as follows: [(HFD animal mean – CTL animal mean)/ CTL animal mean]. The positive or negative value helped us to quickly identify if the metabolite was increased or decrease in CTL or HFD animals. Then, the heatmaps were built by the Metaboanalyst 6.0 software (accessed on 1 March 2024 https://www.metaboanalyst.ca/).

We used them to look for hidden metabolomic pattern in serum, urine, faecal extract, and microbiota sample.

Additionally, to the global metabolome analysis (polar metabolites and lipids) in the liver, serum, urine and faecal extract, a lipid moieties profile was obtained only in the liver and serum samples at t21 to analyse and compare only the lipids in those samples as described in Martin-Grau M *et al* [15]. Briefly, the lipid moieties regions were integrated using MestReNova software (MestReNova v14.1.1, Mestrelab Research S.L, Santiago de Compostela, Spain). A total of 6 regions were considered for this analysis (**Tables S1** and **S2**) and the selected lipid were as follows: saturated fatty acids (SFA), long chain carbonyl groups (lcCO), total carbonyl groups (tCO), long chain unsaturated fatty acids (lcUFA), polyunsaturated fatty acids (PUFA), and total unsaturated fatty acids (tUFA). The data were normalized to the total amount of water of the sample and in the liver spectra, to the liver portion used for its measurement.

S4.5. Statistical analysis and biological interpretation

Statistical analysis was carried out using SPSS software (IBM SPSS Statistics 28.0, New York, USA). First, the evaluation of the normality was performed by the Kolmogorov – Smirnov normality test. For the variables with normal distribution, the parametric tests applied were as follows: Levene test for homogeneity of variances, *Student's t* test for comparison between 2 groups (liver metabolome data at t21 showed in Figure 1), two-way ANOVA for end time measures (only at t21) and comparison of all the groups (anthropometrical data, biochemistry and blood cell count data, histological assessment, lipid moieties profile at t21 from liver and serum metabolomic data), factorial ANOVA for repeated measures and comparison of all the groups (IP GTT data, global metabolomics of serum, urine and faecal samples, and microbiota data). Bonferroni *post hoc* correction tests were applied in data analysed by ANOVA test (two-way or factorial) to compare variables two-by-two. However, for liver metabolomics data, in which *Student's t* test were applied (comparison between 2 groups).

For the variables that did not follow a normal distribution, the non-parametric tests applied were as follows: Friedman for repeated measures (equivalent to factorial ANOVA), Wilcoxon test for pairwise related samples, and Mann-Whitney U test for pairwise independent samples (both equivalent to *post hoc* correction). Only the data from microbiota related to the Lactobacillus genus did not follow a normal distribution and these non-parametric tests were applied.

In addition, intra-subject variability was checked by a linear regression and the housing effect by cage was checked by ANOVA as an additional variable.

We used different symbols to compare CTL vs HFD groups (*), or males vs females (†). Generally, statistical significance was set at $^{*}p<0.05$, $^{**}p<0.01$, or $^{***}p<0.001$. However, for liver metabolomics data (Figure 1), in which *Student's t* test were applied (comparison between 2 groups), adjusted *p_value* was set at $^{*}p<0.00081$, $^{**}p<0.00016$, or $^{***}p<0.000016$. Adjusted *p_value* was calculated as alpha error divided by the total number of metabolites analysed in the liver (0.05/62; 0.01/62; 0.001/62). One CTL male and one HFD female were removed from the study due to kidney damage and alteration of the different studied parameters without being produced by the CTL or HFD diet. They were considered as outlier animals. Thus, the number of animals per group was n=7 in CTL males, n=10 in HFD males, n=8 in CTL females, n=9 in HFD females. For the microbiota study, only an n=4 per group was used.

Supplementary figures and tables

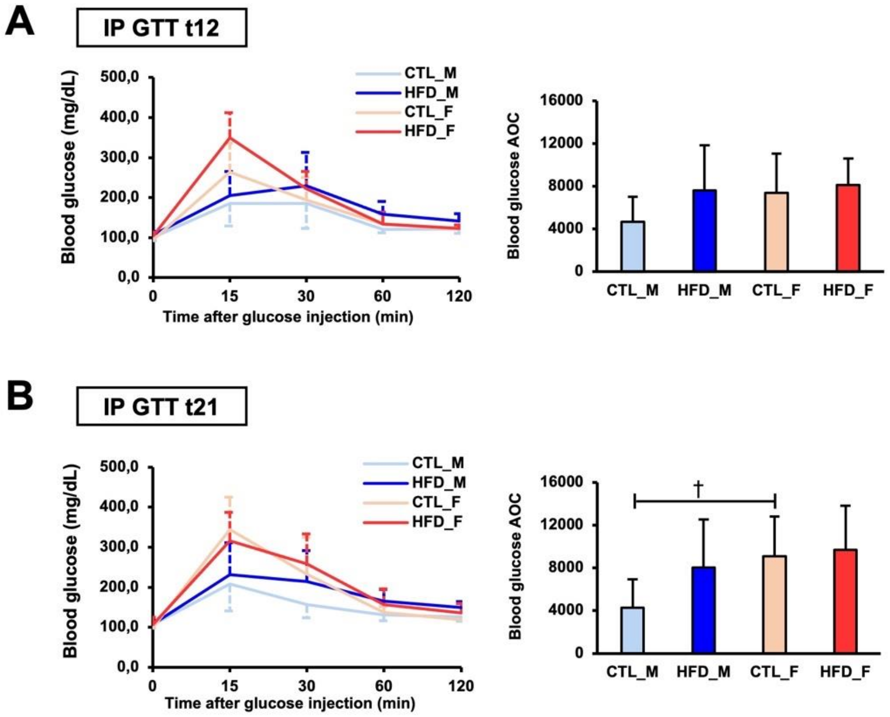

Figure S1. Basal glycaemia assessment in male and female Wistar rats. (A and B) Intraperitoneal glucose tolerance test (IP GTT) in male and female Wistar rats at different experimental time-point after (A) 12 weeks (t12), and after (B) 21 weeks (t21) of diet intervention. Representation of the area of the curve (AOC) from IP GTT appeared next to their graphical results. *n*=7 in CTL males, *n*=10 in HFD males, *n*=8 in CTL females, *n*=9 in HFD females. Statistical significance was set at $^{\dagger}p<0.05$ (males vs females) (factorial ANOVA for repeated measures and Bonferroni *post hoc* test).

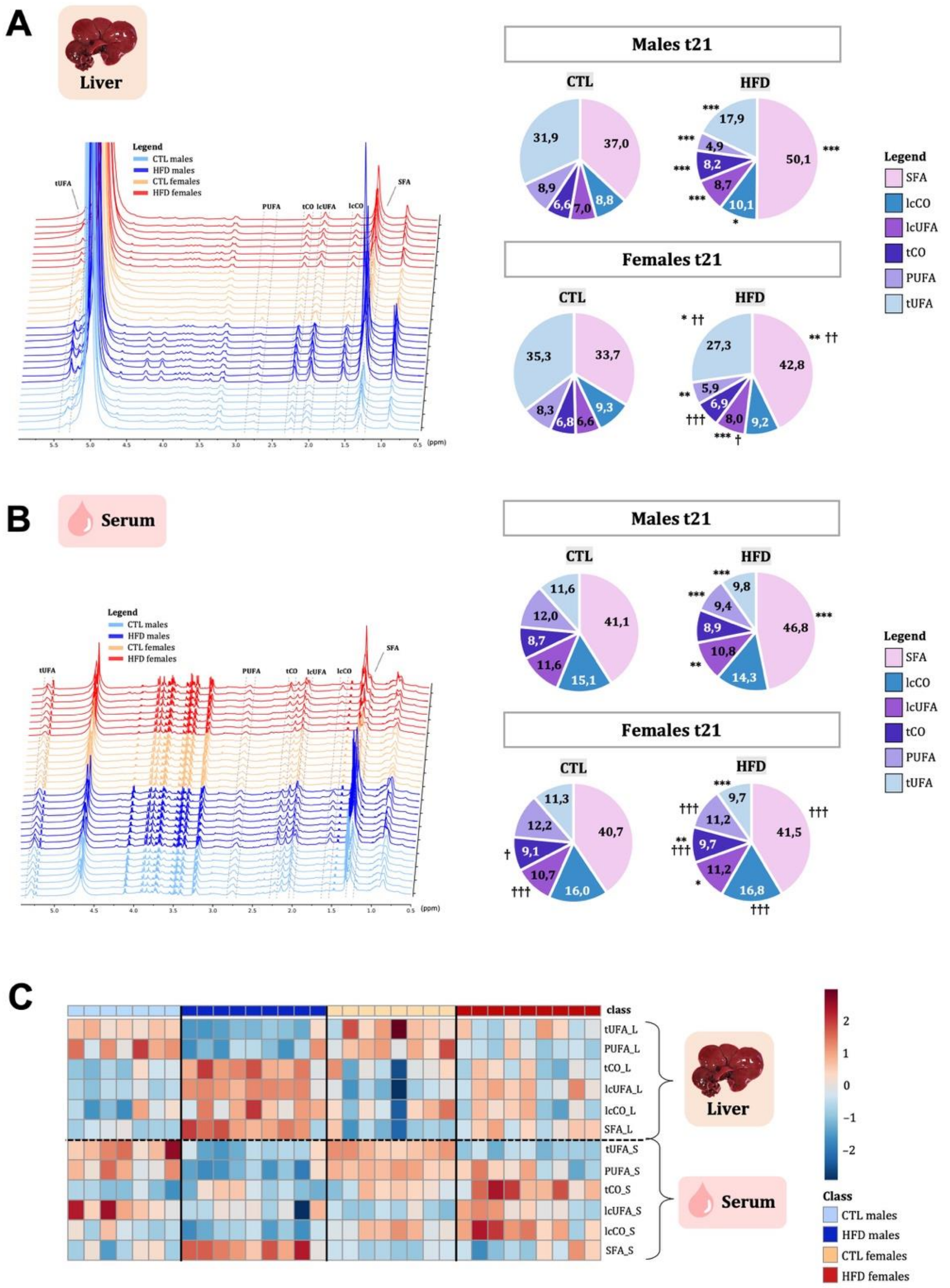

Figure S2. Lipid moieties profile comparisons between liver and serum samples at t21. Profile of lipid moieties in (A) liver tissue and (B) serum at week 21. (A and B) On the left side, the ^{1}H-NMR spectra are observed, and, on the right, the lipid moieties quantification expressed in percentages considering the total sum of lipids. *n*=7 in CTL males, *n*=9 in HFD males, *n*=8 in CTL females, *n*=9 in HFD females. Statistically significant differences (two-way ANOVA and Bonferroni *post hoc* test) were set at **p*<0.05, ***p*<0.01, and ****p*<0.001 (CTL vs HFD groups) or †*p*<0.05, †*p*<0.01, and †*p*<0.001 (males vs females). (C) Heatmap comparing the lipid moieties from the liver and serum at week 21. The colour scale showed the metabolite concentration in each animal condition from highest (red) to lowest (blue). lcCO, long chain carbonyl groups; lcUFA, long chain unsaturated fatty acids; SFA, saturated fatty acids; PUFA, polyunsaturated fatty acids; tCO, total carbonyl groups; tUFA, total unsaturated fatty acids. _S indicated that was measured in serum, while _L indicated that was measured in the liver.

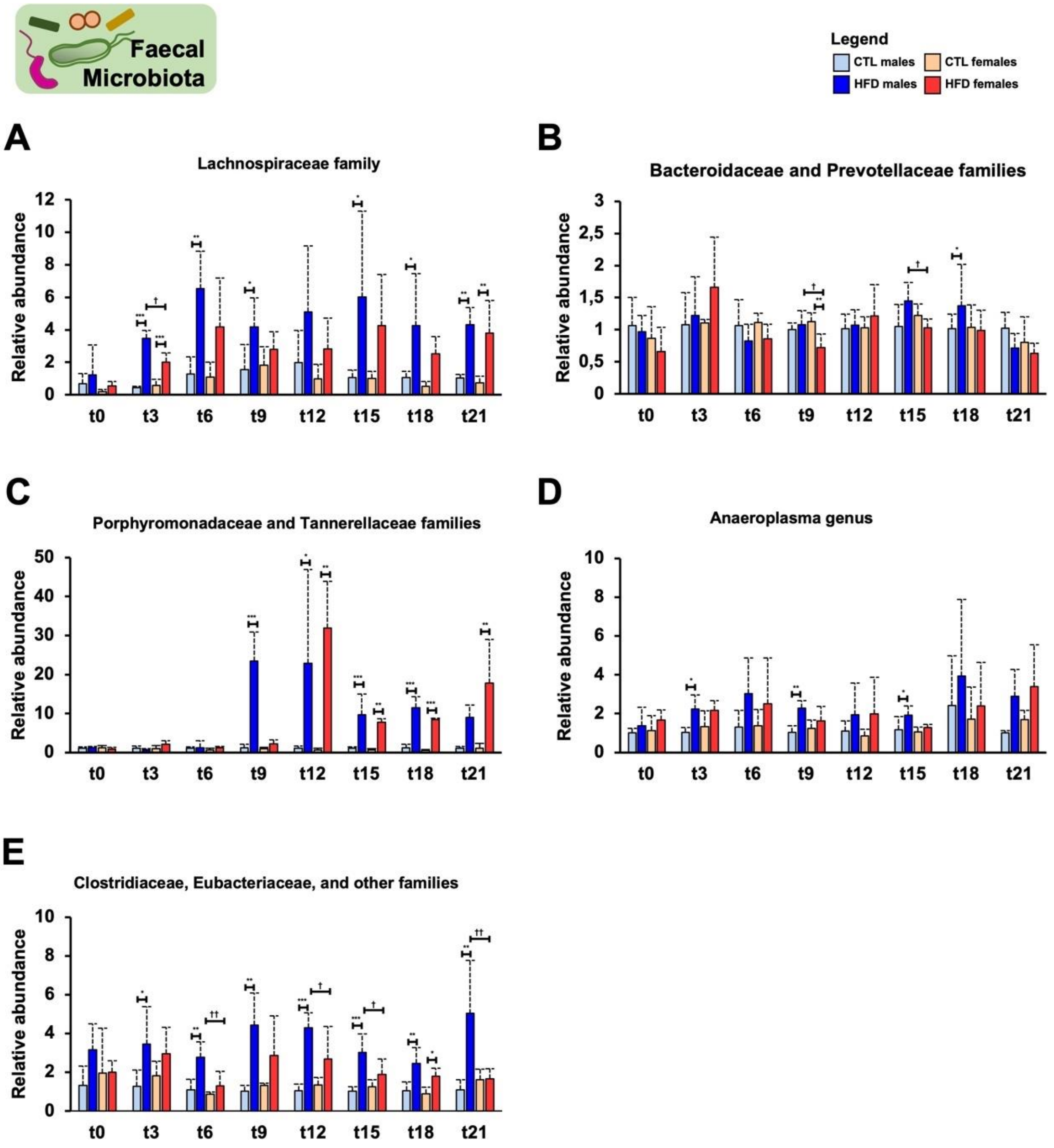

Figure S3. Faecal microbiota data over time and among groups. Relative abundance of (A) Lachnospiraceae family, (B) Bacteroidaceae, and Prevotellaceae families, (C) Porphyromonadaceae and Tannerellaceae families, (D) Anaeroplasma genus, and (E) Clostridiaceae, Eubacteriaceae, and other families. *n*=4 animals per group. Statistical significance was set at **p*<0.05, ***p*<0.01, ****p*<0.001 (CTL vs HFD group) and †*p*<0.05 (males vs females) (factorial ANOVA for repeated measures and Bonferroni *post hoc* test).

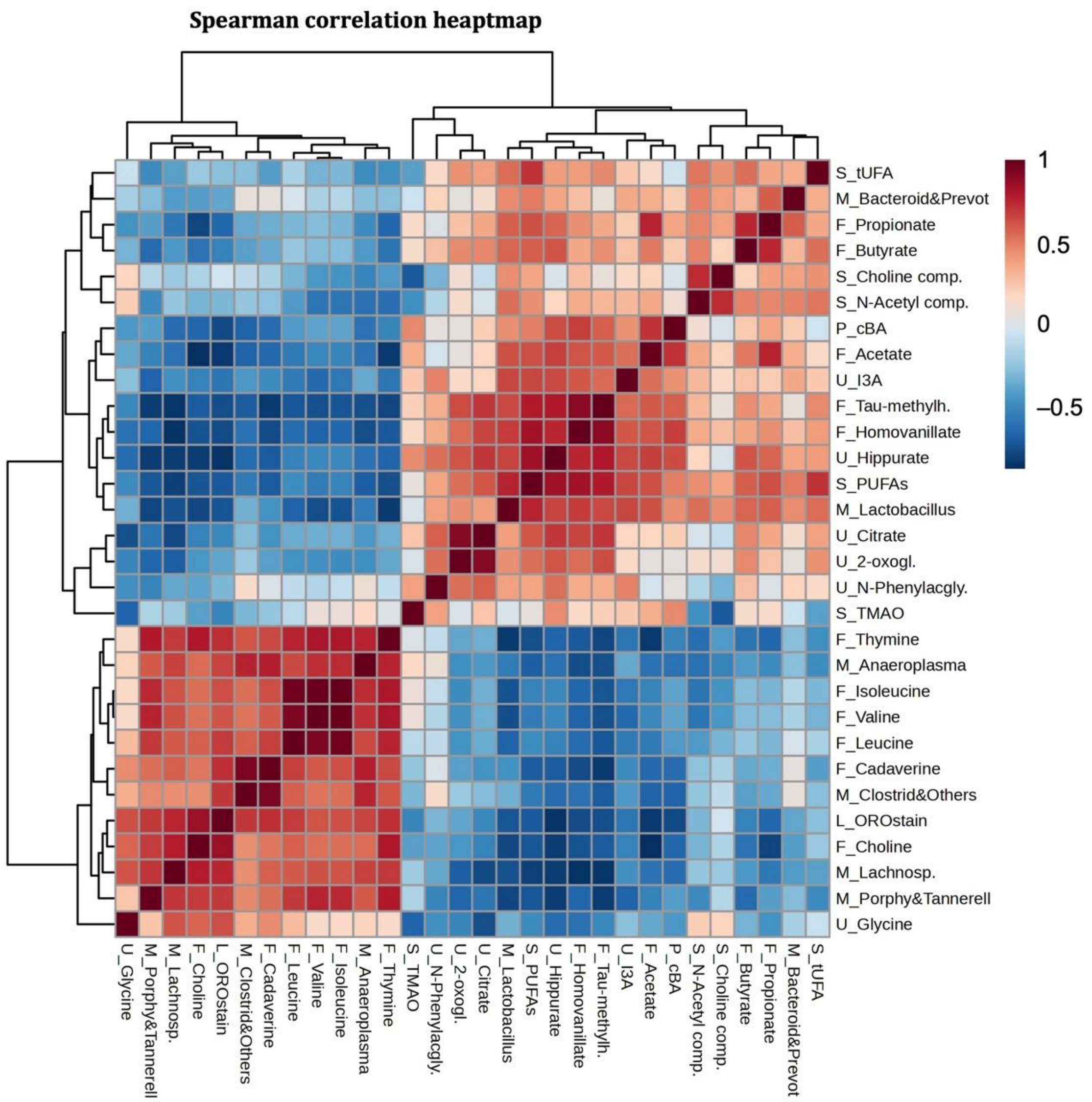

Figure S4. Spearman correlations of the metabolic factors with relative abundance of microbial species at week 21. The factors were abbreviated as M_ for microbiota data, S_ for serum, F_ for faeces, U_ for urine, L_ for liver, and P_ for plasma. Abbreviations: 2-oxogl., 2-oxoglutarate; cBA, circulating bile acids; I3A, Indole-3-acetate; N-Acetyl comp., N-Acetyl compounds; N-Phenylacgly, N-Phenylacetylglycine; ORO, Oils red O stain; PUFA, polyunsaturated fatty acids; Tau-methylh., tau-methylhistidine; TMAO, trimethylamine N-oxide; tUFA, total unsaturated fatty acids.

Table S1. Metabolites in the liver spectra.

Metabolite		^{1}H-NMR Chemical shift (ppm), and multiplicity	HMDB ref.	KEGG ref.
**Acetate**		**1.9 (s)**	HMDB0000042	C00033
**L-Alanine**		**1.5 (d)**, 3.8 (q)	HMDB0000161	C00041
**Alpha-Glucose**		3.2 (t), 3.4 (t), 3.5 (t, dd, m), 3.7 (dd, t), 3.8 (dd, m), 3.9 (dd), 4.6 (d), **5.2 (d)**	HMDB0003345	C00267
**Ascorbate**		3.7 (q), 3.8 (q), 4.0 (m), **4.5 (d)**	-	C00072
**Betaine**		**3.3 (s)**, 3.9 (s)	HMDB0000043	C00719
**Choline compounds**		**3.2**		
**Choline**		**3.2 (s)**, 3.5 (t), 4.1 (m)	HMDB0000097	C00114
**O-Phosphocholine**		**3.2 (s)**, 3.6 (t), 4.2 (m)	HMDB0001565	C00588
**sn-glycero-3-phosphocholine**		**3.2 (s)**, 3.6 (q), 3.7 (dd, t), 3.9 (m), 4.3 (m)	HMDB0000086	C00670
**Fatty acid regions**	**Free cholesterol**	**0.7 (s)**	-	-
	**Lipoparticles**	**0.8 - 0.9**	-	-
	**SFA**	**1.2 - 1.3**	-	-
	**lcCO**	**1.5 - 1.6**	-	-
	**lcUFA**	**2.0**	-	-
	**tCO**	**2.2 - 2.3**	-	-
	**PUFA-Arachidonic acid**	**2.8**	-	-
	**PUFA- Linoleic acid**	**2.7**	-	-
	**PUFA-omega3**	**2.8**	-	-
	**tUFA**	**5.3 - 5.4**	-	-
**Formate**		**8.5 (s)**	HMDB0000142	C00058
**Fructose**		3.5 (d), 3.6 (d), 3.7 (dd, d, q), 3.8 (d, dd, m), 3.9 (dd), **4.0 (s, d)**, 4.1 (d, t)	HMDB0000660	C02336
**Glutathione**		2.1 (q), 2.2 (q), 2.5 (m), 2.6 (m), 2.9 (dd), **3.0 (dd)**, 3.8 (d, dd, t), 4.6 (t), 8.2 (s), 8.5 (s)	HMDB0062697	C00051
**Glycogen**		3.6-4.0, **5.40 (m)**	HMDB0000757	C00182
**Inosine**		3.8 (dd), 3.9 (dd), 4.3 (q), 4.4 (t), 4.8 (t), **6.1 (d)**, 8.2 (s), 8.4 (s)	HMDB0000195	C00294
**L-Isoleucine**		0.9 (t), **1.0 (d)**, 1.2 (m), 1.5 (m), 2.0 (m), 3.7 (d)	HMDB0000172	C00407
**L-Leucine**		0.9 (d), **1.0 (d)**, 1.7 (m), 3,7 (q)	HMDB0000687	C00123
**L-Lysine**		1.4 (m), 1.5 (m), 1.7 (m), 1.9 (m), **3.0 (t)**, 3.7 (t)	HMDB0000182	C00047
**Mannose**		3.4 (m), 3.6 (t), 3.7 (dd, t, q), 3.8 (dd, q, m), 3.9 (d, dd, m), 4.9 (s), **5.2 (d)**	HMDB0000169	C00936
**Methanol**		**3.4 (s)**	HMDB0001875	C00132
**N-Acetyl compounds**		**2.0-2.1**, 7.9-8.1		
**N-acetylcysteine**		**2.1 (s)**, 2.9 (dd), 4.4 (m), 8.0 (s)	HMDB0001890	C06809
**N-acetyl-D-glucosamine**		**2.0 (s)**, 3.4 (t, m), 3.5 (t), 3.7 (dd, q), 3.8 (d, t, q, m), 3.9 (d, m), 4.7 (d), 5.2 (d), 8.1 (d), 8.2 (d)	HMDB0000215	C00140
**N-acetylglutamine**		1.9 (m), **2.0 (s)**, 2.1 (m), 2.3 (m), 4.2 (m), 6.8 (s), 7.5 (s), 7.9 (d)	HMDB0006029	-
**N-acetylglycine**		**2.0 (s)**, 3.7 (d), 8.0 (s)	HMDB0000532	-
**Oxalacetic acid**		**3.3 (s)**	HMDB0000223	C00036
**L-Phenylalanine**		3.1 (q), 3.3 (dd), 4.0 (q), 7.3 (d), **7.4 (t)**	HMDB0000159	C00079
**Phosphatidylcholine**		**4.3**	-	-
**Tau-methylhistidine**		3.1 (q), 3.2 (dd), 3.7 (s), 4.0 (q), 7.0 (s), **7.7 (s)**	HMDB0000001	C01152
**Taurine**		3.3 (t), **3.4 (t)**	HMDB0000251	C00245
**TMAO**		**3.3 (s)**	HMDB0000925	C01104
**L-Tyrosine**		3.0 (q), 3.2 (dd), 3.9 (q), **6.9 (d)**, 7.2 (d)	HMDB0000158	C00082
**Uridine**		3.8 (dd), 3.9 (dd), 4.1 (m), 4.2 (t), 4.3 (t), **5.9 (d)**, 7.9 (d)	HMDB0000296	C00299
**Valine**		**1.0 (d)**, 2.3 (m), 3.6 (d)	HMDB0000883	C00183

The peak/s that were quantified appear in **bold letter [ppm, (multiplicity)]**. Multiplicity in proton NMR: s, singlet; d, doublet; dd, double doublet; t, triplet; q, quartet; m, multiplet (quintet or more). HMDB, human metabolome database; KEGG, Kyoto encyclopaedia of genes and genomes; lcCO, long chain carbonyl groups; lcUFA, long chain unsaturated fatty acids; PUFA, polyunsaturated fatty acids; SFA, saturated fatty acids; tCO, total carbonyl groups; TMAO, trimethylamine N-oxide; tUFA, total unsaturated fatty acids.

Table S2. Metabolites in the serum spectra.

Metabolite		^{1}H-NMR Chemical shift (ppm), and multiplicity	HMDB ref.	KEGG ref.
**Choline compounds**		**3.2**		
Choline		**3.2 (s)**, 3.5 (t), 4.1 (m)	HMDB0000097	C00114
O-Phosphocholine		**3.2 (s)**, 3.6 (t), 4.2 (m)	HMDB0001565	C00588
sn-glycero-3-phosphocholine		**3.2 (s)**, 3.6 (q), 3.7 (dd, t), 3.9 (m), 4.3 (m)	HMDB0000086	C00670
	**Free cholesterol**	**0.7 (s)**	-	-
	**Lipoparticles**	**0.8 - 0.9**	-	-
	**SFA**	**1.2 - 1.3**	-	-
**Fatty acid regions**	**lcCO**	**1.5 - 1.6**	-	-
	**lcUFA**	**2.0**	-	-
	**tCO**	**2.2 - 2.3**	-	-
	**PUFA**	**2.7 - 2.8**	-	-
	**tUFA**	**5.3 - 5.4**	-	-
**N-Acetyl compounds**		**2.0 - 2.1**		
N-acetyl-L-aspartate		**2.0 (s)**, 2.5 (q), 2.7 (dd), 4.4 (m), 7.9 (d)	HMDB0000812	C01042
N-acetylcysteine		**2.1 (s)**, 2.9 (dd), 4.4 (m), 8.0 (s)	HMDB0001890	C06809
N-acetylglutamate		1.9 (m), **2.0 (s, m)**, 2.2 (t), 4.1 (m), 8.0 (d)	HMDB0001138	C00624
N-acetylglutamine		1.9 (m), **2.0 (s)**, 2.1 (m), 2.3 (m), 4.2 (m), 6.8 (s), 7.5 (s), 7.9 (d)	HMDB0006029	-
N-acetylglycine		**2.0 (s)**, 3.7 (d), 8.0 (s)	HMDB0000532	-
N-acetylornithine		1.7 (m), 1.8 (m), **2.0 (s)**, 3.0 (t), 4.2 (m), 8.0 (d)	HMDB0003357	C00437
**TMAO**		**3.3 (s)**	HMDB0000925	C01104

The peak/s that were quantified appear in **bold letter [ppm, (multiplicity)]**. Multiplicity in proton NMR: s, singlet; d, doublet; dd, double doublet; t, triplet; q, quartet; m, multiplet (quintet or more). HMDB, human metabolome database; KEGG, Kyoto encyclopaedia of genes and genomes; lcCO, long chain carbonyl groups; lcUFA, long chain unsaturated fatty acids; PUFA, polyunsaturated fatty acids; SFA, saturated fatty acids; tCO, total carbonyl groups; TMAO, trimethylamine N-oxide; tUFA, total unsaturated fatty acids.

Table S3. Metabolites in the urine spectra.

Metabolite	^{1}H-NMR Chemical shift (ppm), and multiplicity	HMDB ref.	KEGG ref.
**2-oxoglutarate**	**2.4 (t)**, 3.0 (t)	HMDB0000208	C00026
**Citrate**	**2.5 (d)**, 2.7 (d)	HMDB0000094	C00158
**Glycine**	**3.6 (s)**	HMDB0000123	C00037
**Hippurate**	4.0 (d), 7.5 (t), **7.6 (t)**, 7.8 (d), 8.5 (s)	HMDB0000714	C01586
**Indole-3-acetate**	3.6 (s), 7.2 (s, t), **7.3 (t)**, 7.5 (d), 7.6 (d), 10.0 (s)	HMDB0000197	C00954
**N-Phenylacetylglycine**	3.7 (s, d), **7.3 (d)**, 7.4 (t), 8.0 (s)	HMDB0000821	C05598

The peak/s that were analysed appear in **bold letter [ppm, (multiplicity)]**. Multiplicity in proton NMR: s, singlet; d, doublet; t, triplet. HMDB, human metabolome database; KEGG, Kyoto encyclopaedia of genes and genomes.

Table S4. Metabolites in the faecal spectra.

Metabolite	^{1}H-NMR Chemical shift (ppm), and multiplicity	HMDB ref.	KEGG ref.
**Acetate**	**1.9 (s)**	HMDB0000042	C00033
**Butyrate**	**0.9 (t)**, 1.5 (m), 2.1 (t)	HMDB0000039	C00246
**Cadaverine**	1.5 (m), 1.7 (m), **3.0 (t)**	HMDB0002322	C01672
**Choline**	**3.2 (s)**, 3.5 (t), 4.1 (m)	HMDB0000097	C00114
**Homovanillate**	3.4 (s), 3.9 (s), **6.7 (dd)**, 6.9 (d)	HMDB0000118	C05582
**L-Isoleucine**	0.9 (t), **1.0 (d)**, 1.2 (m), 1.5 (m), 2.0 (m), 3.7 (d)	HMDB0000172	C00407
**L-Leucine**	**0.9 (d)**, 1.0 (d), 1.7 (m), 3,7 (q)	HMDB0000687	C00123
**Propionate**	**1.0 (t)**, 2.2 (q)	HMDB0000237	C00163
**Tau-methylhistidine**	3.1 (q), 3.2 (dd), 3.7 (s), 4.0 (q), **7.0 (s)**, 7.7 (s)	HMDB0000479	C01152
**Thymine**	**1.9 (s)**, 7.4 (s)	HMDB0000262	C00178
**L-Valine**	**1.0 (d)**, 2.3 (m), 3.6 (d)	HMDB0000883	C00183

The peak/s that were analysed appear in **bold letter [ppm, (multiplicity)]**. Multiplicity in proton NMR: s, singlet; d, doublet; dd, double doublet; td, triple doublet; t, triplet; dt, double triplet; q, quartet; m, multiplet (quintet or more). HMDB, human metabolome database; KEGG, Kyoto encyclopaedia of genes and genomes.

Table S5. 16S rRNA gene-targeted group-specific primers for microbial detection.

16S rRNA primers used in qPCR assay	Primer sequence (5'-3')	AS (bp)	AT (°C)	Ref.
**Universal 16S rRNA 520F-799R**	F: AGCAGCCGCGGTAATACG<br>R: CAGGGTATCTAATCCTGTTCG	270	58	Redesigned from [27]
**Bacteroidaceae and Prevotellaceae families**	F: GAGAGGAAGGTCCCCCAC<br>R: CGCTACTTGGCTGGTTCAG	108	58	From [28]
**Porphyromonadaceae and Tannerellaceae families**	F: AAATCAGCACGGCCCTTACA<br>R: CAGCTTCACGGAGTCGAGTT	141	60	This study
***Lactobacillus* genus**	F: GAGGCAGCAGTAGGGAATCTTC<br>R: GGCCAGTTACTACCTCTATCCTTC	126	60	From [26]
**Lachnospiraceae family**	F: ACACGTGCTACAATGGCGTA<br>R: ACTGACTTCGGGCGTTACTG	214	60	This study
**Clostridiaceae, Eubacteriaceae, and other families**	F: CGCACAAGCAGTGGAGT<br>R: ACCTTCCTCCGTTTTGTCAA	249	57	Redesigned from [26]
***Anaeroplasma* genus**	F: CCTGGGCTACAAACGTGCTA<br>R: GTACAAAGCCCGRGAACGTA	179	59	This study

AS, amplicon size; AT(°C), annealing temperature; bp, base pair; F: forward primer; R: reverse primer.

Table S6. Target species detected by 16S rRNA group-specific primers.

Phylum	Groups	Target species*
**Bacteroidetes**	**Bacteroidaceae and prevotellaceae families**	**Bacteroidaceae family**<br>*Bacteroides propionicigenes, B. humanifaecies, B. luhongzhouii, B.zhangwenhongii, B.faecalis, B.cutis, B.togonis, B.ilei, B.ndongoniae, B.congonensis, B.bouchesdurhonensis, B.sedimenti, B.neonati, B.kribbi, B.fragilis, B.koreensis, B.caecicola, B.ihuae, B.mediterraneensis, B.caecimuris, B.caecigallinarum, B.stercorirosoris, B.faecichinchillae, B.zoogleoformans, B.rodentium, B.oleiciplenus, B.graminisolvens, B.fluxus, B.faecis, B.clarus, B.rodentium, B.pyogenes, B.xylanisolvens, B.uniformis, B.thetaiotaomicron, B.stercoris, B.salyersiae, B.ovatus, B.nordii, B.helcogenes, B.eggerthii, B.cellulosilyticus, B.caccae, B.acidifaciens, B.luti, B.timonensis, B.reticulotermitis, B.acidifaciens, B.oleiciplenus, B.clarus, B.gallinarum, B.intestinalis, B.finegoldii, B.cellulosilyticus, B.helcogenes, B.heparinolyticus, B.salyersiae, Caecibacteroides pullorum, Mediterranea massiliensis, Paraphocaeicola brunensis, Phocaeicola faecalis, P.faecicola, P.paurosaccharolyticus, P.sartorii, P.vulgatus, P.massiliensis, P.coprophilus, P.barnesiae, P.dorei, P.plebeius, P.coprocola*<br><br>**Prevotellaceae family**<br>*Alloprevotella rava, A.tannerae, Halella colorans, H.seregens, Hoylesella nanceiensis, H.marshii, H.loescheii, H.enoeca, H.buccalis, H.pleuritidis, H.shahii, Leyella stercorea, Massiliprevotella massiliensis, Paraprevotella xylaniphila, P.clara, Prevotella illustrans, P.hominis, P.brunnea, P.marseillensis, P.phocaeensis, P.lacticifex, P.cerevisiae, P.rara, P.herbatica, P.mizrahii, P.vespertina, P.ihumii, P.saccharolytica, P.oryzae, P.veroralis, P.timonensis, P.paludivivens, P.pallens, P.oulorum, P.oris, P.oralis, P.nigrescens, P.melaninogenica, P.maculosa, P.intermedia, P.histicola, P.disiens, P.dentasini, P.denticola, P.dentalis, P.corporis, P.bivia, P.amnii, P.albensis, P.melaninogenica, P.ruminicola, P.jejuni, P.scopos, P.fusca, P.oulorum, P.aurantiaca, P.intermedia, P.falsenii, P.marshii, P.multiformis, P.salivae, P.disiens, P.buccalis, P.brevis, P.bryantii, P.pallens, Prevotellamassilia timonensis, Pseudoprevotella muciniphila, Segatella copri*
	**Porphyromona-daceae and Tannerellaceae families**	**Porphyromonadaceae family**<br>*Porphyromonas pasteri, P.gingivicanis, P.gingivalis, P.pogonae, P.gulae, P.endodontalis, P.circumdentaria, P.catoniae, P.cangingivalis, P.loveana, Macellibacteroides fermentans, Massilibacteroides vaginae*

		**Tannerellacea family**<br>*Parabacteroides acidifaciens, P.pacaensis, P. provencensis, P.chinchillae, P.chartae, P.johnsonii, P.merdae, Tannerella serpentiformis, T.forsythia,*
**Firmicutes**	**Lactobacillus genus**	*Lactobacillus mulieris, L.corticis, L.xujianguonis, L.crispatus, L.helveticus, L.acidophilus, L.gallinarum, L.jensenii, L.intestinalis, L.amylolyticus, L.hamsteri, L.kefiranofaciens, L.ultunensis, L.amylovorus, L.psittaci, L.kitasatonis, L.ultunensis, L.fornicalis*
	**Lachnospira-ceae family**	*Anaerostipes hominis, A.faecalis, A.rhamnosivorans, A.hadrus, A.butyraticus, A.caccae, Bariatricus massiliensis, Blautia hominis, B.argi, B.marasmi, B.intestinalis, B.provencensis, B.phocaeensis, B.pseudococcoides, B.luti, B.producta, B.glucerasea, B.coccoides, B.hansenii, B.wexlerae, B.schinkii, B.hydrogenotrophica, B.obeum, Coprococcus phoceensis, Dorea phocaeensis, D.formicigenerans, Faecalimonas umbilicate, Hespellia stercorisuis, H.porcina, Lachnoclostridium symbiosum, Mediterraneibacter massiliensis, Ruminococcus faecis, R.gnavus, R.torques, R.lactaris, Murimonas intestine, Qiania dongpingensis, Sporobacterium olearium, Clostridium nexile*
	**Oscillospiraceae, Clostridiaceae, and other families**	**Oscillospiraceae family**<br>*Acetanaerobacterium elongatum, Acutalibacter muris, Agathobaculum butyriciproducens, A. desmolans, Anaeromassilibacillus senegalensis, Anaerotruncus massiliensis, A.colihominis, A. rubiinfantis, Angelakisella massiliensis, Bittarella massiliensis, Caproicibacter fermentans, Caproicibacterium lactatifermentans, C. amylolyticum, Caproiciproducens galactitolivorans, Ethanoligenens harbinense, Faecalibacterium hattorii, F.longum, F.butyricigenerans, F.gallinarum, F.duncaniea, F.prausnitzii, Fournierella massiliensis, Fumia xinanensis, Gorbachella massiliensis, Harryflintia acetispora, Hydrogeniiclostridium mannosilyticum, Marasmitruncus massiliensis, Massiliimalia timonensis, M.massiliensis, Neglectibacter timonensis, Papillibacter cinnamivorans, Paludihabitans psychrotolerans, Provencibacterium massiliense, Pseudoruminococcus massiliensis, Pygmaiobacter massiliensis, Ruminococcoides bili, Ruminococcus bovis, R.albus, R. callidus, R. champanellensis, R. bromii, R.flavefaciens, Ruthenibacterium lactatiformans, Scatolibacter rhodanostii, Solibaculum mannosilyticum, Subdoligranulum variabile, Tepidibaculum saccharolyticum, Clostridium leptum, Clostridium methylpentosum, Eubacterium siraeum*<br><br>**Clostridiaceae family**<br>*Butyricicoccus intestinisimiae, B.porcorum, B.faecihominis, B. pullicaecorum, Clostridium minihomine, C. merdae, C. jeddahense, Massilioclostridium coli*<br><br>**Eubacteriaceae family**<br>*Intestinibacillus massiliensis*<br><br>**Feifaniaceae family**<br>*Feifania hominis*<br><br>**Yeguiaceae family**<br>*Yeguia hominis*<br><br>**Eubacteriales incertae sedis (no rank)**<br>*Gemmiger gallinarum, G.formicilis,*
**Tenericutes**	**Anaeroplasma genus**	*Anaeroplasma bactoclasticum, A.varium, A.abactoclasticum*

* Target species were obtained from PRIMER Blast Database introducing the forward and reverse primers.
